# Linking functional composition moments of the sub-Mediterranean ecotone with environmental drivers

**DOI:** 10.3389/fpls.2023.1303022

**Published:** 2023-12-08

**Authors:** Sergio de Tomás Marín, Javier Galán Díaz, Jesús Rodríguez-Calcerrada, Iván Prieto, Enrique G. de la Riva

**Affiliations:** ^1^ Department of Ecology, Brandenburgische Technische Universität Cottbus-Senftenberg, Cottbus, Germany; ^2^ Department of Pharmacology, Pharmacognosy and Botany, Faculty of Pharmacy, Universidad Complutense de Madrid, Madrid, Spain; ^3^ Functioning of Forest Systems in a Changing Environment Research Group, Universidad Politécnica de Madrid, Madrid, Spain; ^4^ Ecology Department, Faculty of Biology and Environmental Sciences, Universidad de León, León, Spain

**Keywords:** community assembly, community-weighted moments, ecotone, functional structure, intraspecific variability, mixed-species forest, sub-Mediterranean community, trait-based

## Abstract

**Introduction:**

Functional trait-based approaches are extensively applied to the study of mechanisms governing community assembly along environmental gradients. These approaches have been classically based on studying differences in mean values among species, but there is increasing recognition that alternative metrics of trait distributions should be considered to decipher the mechanisms determining community assembly and species coexistence. Under this framework, the main aim of this study is to unravel the effects of environmental conditions as drivers of plant community assembly in sub-Mediterranean ecotones.

**Methods:**

We set 60 plots in six plant communities of a sub-Mediterranean forest in Central Spain, and measured key above- and belowground functional traits in 411 individuals belonging to 19 species, along with abiotic variables. We calculated community-weighted mean (CWM), skewness (CWS) and kurtosis (CWK) of three plant dimensions, and used maximum likelihood techniques to analyze how variation in these functional community traits was driven by abiotic factors. Additionally, we estimated the relative contribution of intraspecific trait variability and species turnover to variation in CWM.

**Results and discussion:**

The first three axes of variation of the principal component analyses were related to three main plant ecological dimensions: Leaf Economics Spectrum, Root Economics Spectrum and plant hydraulic architecture, respectively. Type of community was the most important factor determining differences in the functional structure among communities, as compared to the role of abiotic variables. We found strong differences among communities in their CWMs in line with their biogeographic origin (Eurosiberian vs Mediterranean), while differences in CWS and CWK indicate different trends in the functional structure among communities and the coexistence of different functional strategies, respectively. Moreover, changes in functional composition were primarily due to intraspecific variability.

**Conclusion:**

We observed a high number of strategies in the forest with the different communities spreading along the acquisitive-conservative axis of resource-use, partly matching their Eurosiberian-Mediterranean nature, respectively. Intraspecific trait variability, rather than species turnover, stood as the most relevant factor when analyzing functional changes and assembly patterns among communities. Altogether, our data support the notion that ecotones are ecosystems where relatively minor environmental shifts may result in changes in plant and functional composition.

## 1 Introduction

**Figure 1:**
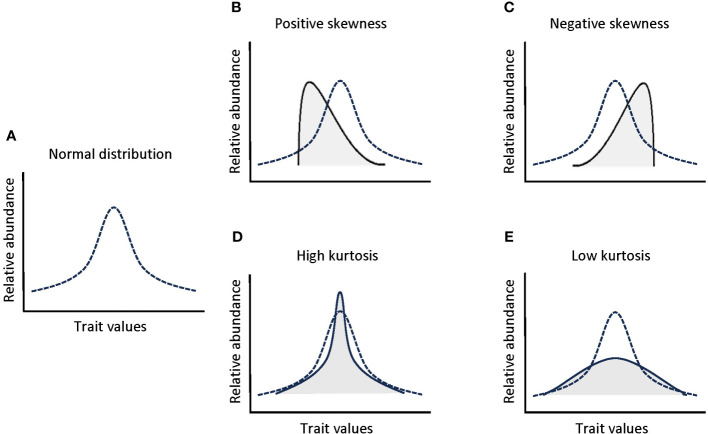
Schematic representation of the shifts in the shape of a normal trait distribution **(A)** produced by skewness (**B**: positive; **C**: negative) and kurtosis (**D**: high; **E**: low).

The functional trait-based approach is a broadly-known tool applied to the study of mechanisms governing community assembly along environmental gradients (Garnier and Navas, 2012; de la Riva et al., 2018a; Dong et al., 2020; Kermavnar et al., 2022), being the initial step towards developing a more mechanistic understanding of how the environment influences ecosystem structure and function (Wieczynski et al., 2019). The classical approach for assessing the assembly processes within a given community have mostly aimed at quantifying the trait composition of plant communities, i.e., the presence of species with particular trait values within communities (e.g. Maire et al., 2012; de la Riva et al., 2016a; Hejda et al., 2019) by focusing on the first two moments of a trait distribution weighted by species abundance, i.e. community-weighted mean and variance of their trait distributions (Garnier et al., 2004; Violle et al., 2012). Mean and variance trait values are only meaningful when traits have a normal (gaussian) distribution within the community. However, the distribution of trait values within communities often deviates from the symmetric normal distribution, for example as a result of rapid environmental changes or asymmetric competition favoring a limited portion of the community with specific trait values (Enquist et al., 2015; Le Bagousse-Pinguet et al., 2017; Gross et al., 2021). Thus, there is an increasing recognition that alternative aspects of trait distributions should be considered to decipher the mechanisms determining community assembly and species coexistence, such as the third and fourth community-weighted moments (skewness and kurtosis) (Le Bagousse-Pinguet et al., 2017; Danet et al., 2018; Wieczynski et al., 2019; Gross et al., 2021). The community-weighted skewness and kurtosis bring light on the shape of trait distributions in naturally assembled communities (**Figure 1**) (Gross et al., 2017; Le Bagousse-Pinguet et al., 2021). The skewness quantifies the asymmetry of the trait distribution, where extreme skewness values are found when rare species within a community have infrequent trait values (Le Bagousse-Pinguet et al., 2021). The kurtosis, in turn, is a measure of the evenness of trait distributions, where extreme negative kurtosis values reflect an even distribution of trait values in the community or even bimodal trait distributions, and extreme positive kurtosis values reflect a reduced range of trait values (Gross et al., 2021; Le Bagousse-Pinguet et al., 2021). Thus, combining the skewness and kurtosis of a trait distribution with the mean value for a given trait, provides a more complete understanding of how environmental changes shape the functional diversity and species assembly of plant communities (Wieczynski et al., 2019; Gross et al., 2021). Few works have combined the study of abundance-weighted trait moments, i.e. community weighted mean, skewness and kurtosis, for a wide range of traits at the whole plant level, together with potential environmental drivers simultaneously (Wieczynski et al., 2019), despite the importance of looking simultaneously at multiple traits, since different plant organs may respond differently to different selection pressures (Carvajal et al., 2019). In fact, to the best of our knowledge, this approach has never been performed in ecotones or with belowground traits, as it is done in the present study.

The variation of the indices describing the position of the dominant trait values in the trait space and shape of the trait distribution do not reflect the source of variation along environmental gradients. That is, changes in the functional structure of plant communities may arise due to the replacement of species (species turnover) and/or intraspecific trait variability (ITV). These two sources of variation may contribute in different degrees to the overall change in the functional structure of plant communities (Albert et al., 2010; de Bello et al., 2011; Pérez-Ramos et al., 2012; de la Riva et al., 2016a). Hence, the estimation of the trait distribution moments at the whole-plant level, i.e. including both above- and belowground traits, and the role of species turnover and ITV in the variation of these distribution moments among communities, may reveal different responses of plants to environmental changes, offering higher insights into the assembly processes taking place in communities along environmental gradients (Lepš et al., 2011; Kichenin et al., 2013; de la Riva et al., 2016a).

Ecotones are transitional areas between adjacent biocenosis with high levels of biodiversity, as they host species from the adjacent biocenosis as well as unique species endemic to the ecotone (Hufkens et al., 2009; Vila-Viçosa et al., 2020b; Shea et al., 2022). Ecotones are of especial interest for conservation (Carlson et al., 2014; Rawal et al., 2018; Lopes et al., 2020) for their ecological uniqueness (Danso Marfo et al., 2019), and for contributing to stabilize adjacent ecosystems (Pogue and Schnell, 2001). Further, community assembly processes in ecotones are very dynamic, as slight modifications in environmental conditions lead to substantial changes in plant composition (Ruiz-Labourdette et al., 2012; Brice et al., 2020), which makes them ideal study systems to test such processes (Rahman et al., 2021). However, understanding which environmental factors govern the process of species assembly in transitional areas remains challenging (Kitagawa et al., 2020; Rahman et al., 2021) and could be key to understanding how these communities will respond to future climatic conditions.

Sub-Mediterranean areas are transition areas between Mediterranean and temperate climates. The Iberian Peninsula, in the south of Europe, represents one of the largest ecotones of this kind (Sánchez de Dios et al., 2009), being the low-latitudinal edge for the distribution of many central European species (Gil et al., 2010; Dorado-Liñán et al., 2017; Hernández-Lambraño et al., 2021). Sub-Mediterranean regions host Mediterranean and Eurosiberian species as well as many endemic species (Vila-Viçosa et al., 2020a; Box, 2021; Cantoral et al., 2023), creating a mosaic of unique plant communities with contrasting assembly processes (de la Riva et al., 2023). Sub-Mediterranean regions in the Iberian Peninsula are very sensitive to slight environmental alterations (Sánchez de Dios et al., 2009; Vila-Viçosa et al., 2020a), which makes them highly vulnerable to ongoing climatic change and thus critical areas for conservation (Benito Garzón et al., 2008; Cantoral et al., 2023). Understanding trait-based assembly rules in sub-Mediterranean regions can be critical for understanding how species coexist in these and other ecotones, which could be important to predict the trajectory of ecotone plant communities facing increasingly warmer and drier conditions (Benito Garzón et al., 2008; Frenette-Dussault et al., 2013; Pereira et al., 2021).

The broad aim of this study is to unravel the effects of environmental conditions as drivers of the assembly of plant communities in a sub-Mediterranean forest in Central Spain. To this end, we measured four leaf, two stem and three root traits in a total of 411 individuals of 19 woody plant species in six plant communities. We then calculated three trait distribution moments weighted by the relative abundance of each species in each community, i.e., the community-weighted mean (CWM), skewness (CWS) and kurtosis (CWK), of major axes of variation at the aboveground (i.e. Leaf Economics Spectrum or LES, Wright et al., 2004) and belowground levels (i.e. Root Economics Spectrum or RES; Prieto et al., 2015) across plant communities, and assessed their relationship with environmental variables to estimate which, and to what extent, environmental drivers rule the assembly of sub-Mediterranean plant communities. Additionally, we estimated the proportion of variation in CWMs attributable to species turnover and to ITV to have a detailed view of assembly processes occurring in these communities. Using this novel approach, we will gain a great understanding on the functional structure of sub-Mediterranean plant communities and, ultimately, will be able to guide conservation efforts to maintain their proper functioning (Walker, 1995).

## 2 Materials and methods

### 2.1 Study area

The study was conducted in the ‘Hayedo de Montejo’ (Montejo hereafter), a 125 ha. forest located in the ‘Sistema Central’ range in Spain (41°7’N, 3°30’W), between 1250 and 1550 m a. s. l. Orientation is predominantly northeast, although it ranges from south to north in some locations. Slope varies from nearly flat at higher elevations to ca. 58% in the steepest areas. Soil depth ranges from 30 cm in upper areas to 150 cm down in the valley. The dominant bedrock is micaceous gneiss and soils on top are acidic with a sandy-loam texture (López Santalla et al., 2003; Gil et al., 2010). The climate is sub-Mediterranean, with Mediterranean influence in the east and a continental climate in the west. Mean annual precipitation was 858.8 mm and mean annual temperature was 9.7°C for the period 1994-2021 (Montejo meteorological station). Summers are hot with a marked dry period between July and August, typical of Mediterranean climate.

The Hayedo de Montejo is a sub-Mediterranean, mixed beech-oak forest (*Fagus sylvatica-Quercus pyrenaica*), with a high diversity of plant species and communities coexisting in a relatively small area. In this forest, Eurosiberian species coexist with typically Mediterranean species in the overstory and the understory (Sánchez de Dios et al., 2009; Gil et al., 2010; Rivas-Martínez et al., 2011), which harbors some herbaceous species of special interest for conservation for their rarity, such as *Paris quadrifolia* L., *Narcissus pseudonarcissus* ssp. *pseudonarcissus* L. or *Aconitum napellus* L. (Hernández Bermejo et al., 1982; Moreno et al., 2005; Gil et al., 2010). The forest is dominated by the temperate tree species European beech (*Fagus sylvatica* L.; hereafter beech) and sessile oak (*Quercus petraea* (Matt.) Liebl.), both at the southernmost limit of their distribution range, and the Mediterranean Pyrenean oak (*Quercus pyrenaica* Willd.), at the core of its distribution range (Rubio-Cuadrado et al., 2020; Rubio-Cuadrado et al., 2021; de Tomás Marín et al., 2023).

This forest has been traditionally managed as a wooded pasture (Pardo and Gil, 2005). After centuries of forest exploitation, mainly for firewood and cattle grazing, these traditional land uses ceased in the early 1960s (López Santalla et al., 2003; Pardo and Gil, 2005) – although, today, it is still relatively easy to find a few cows grazing in the forest. These land-use changes transformed the former open woodland structure with dispersed trees and scarce tree recruitment into the dense secondary forest that we find today (Pardo and Gil, 2005; Rubio-Cuadrado et al., 2021). The Hayedo de Montejo forest is included in the ‘Ancient and Primeval Beech Forests of the Carpathians and Other Regions of Europe’ list of European beech forests included in the UNESCO World Heritage Site list. Furthermore, it is embedded in the ‘Sierra del Rincón’ (Rincón mountain range), designated as a Reserve of the Biosphere by UNESCO in 2005.

### 2.2 Sampling design

Three forest inventories (FI) were carried out in Montejo in the years 1994, 2005 and 2015, respectively. In each FI, 125 circular plots of 30-m diameter were systematically established in a 100 x 100 m square grid covering the entire forest. Based on the last FI, we divided the forest into six different areas (communities hereafter) according to the distribution of the three most abundant tree species, i.e., beech, Pyrenean oak and sessile oak (see also de la Riva et al., 2023). An additional community dominated by shrubs where trees are scarce and dispersed was included. These communities were classified based on the abundance of the three main species recorded in the FI of 2015 as follows: (I) *Fagus sylvatica*, (II) *Quercus pyrenaica* or (III) *Quercus petraea*, when >60% of the abundance in terms of tree density corresponded to one of these three species, respectively; (IV) mixed forest 1 (transition areas between *F. sylvatica* and *Q. pyrenaica*), (V) mixed forest 2 (transition area with co-dominance of *F. sylvatica*, *Q. pyrenaica* and *Q. petraea*) and (VI) a shrubland community (**Supplementary Material, Appendix S1, Figure S1**).

Ten random plots from the FIs within each plant community (I to VI) were selected for sampling. In each plot, four parallel 20-m-long transects perpendicular to the slope were randomly set, two on each side of the plot, with a minimum distance of 2 m between transects. Species composition (i.e. identity) and abundance (i.e. % cover) were recorded for each woody species intercepted by the transect line (abundance was measured as the length in meters of canopy of each woody species projecting on the transect line; total abundance may thus exceed 20 m due to overlaying vegetation layers). For leaf, stem and root trait measurements, we selected five individuals per species and community, except for Huber values, where n=3 (see below for description of this trait). Traits were measured in species that together made up at least 90% of the maximum cumulative cover within a community. With this criterion, a total of 19 species were selected across communities: *Adenocarpus hispanicus, Crataegus monogyna, Cytisus purgans, Cytisus scoparius, Erica arborea, Erica australis, Fagus sylvatica, Genista florida, Hedera helix, Ilex aquifolium, Juniperus communis, Lavandula stoechas, Prunus avium, Quercus petraea, Quercus pyrenaica, Rosa* sp.*, Rubus* sp.*, Sorbus aria* and *Sorbus aucuparia*. All species occurred at least in two plant communities, if not more, except *Cytisus purgans*, that was found only in the shrubland community (**Supplementary Material, Appendix S2, Table S2**).

### 2.3 Plant sampling and trait measurements

Plant sampling was carried out in June and July 2021, during the peak of the growing season. From this material, five aboveground and three belowground functional traits related to water and nutrient acquisition were measured (see functional roles in **Table 1**): Leaf Dry Matter Content (LDMC; leaf dry mass per unit of water-saturated fresh mass; mg g^-1^), Specific Leaf Area (SLA; leaf area per unit of leaf dry mass; m^2^ kg^-1^), leaf carbon isotopic composition (δ^13^C; ‰), leaf carbon to nitrogen ratio (leaf C:N), Stem Dry Matter Content (SDMC; stem dry mass per unit of water-saturated fresh mass; mg g^-1^), Huber value (Hv; sapwood cross-sectional branch area to supported leaf area ratio; cm^2^ cm^-2^), Specific Root Area (SRA; root area per unit of root dry mass; m^2^ kg^-1^), average root diameter (Rdi; mm), and root C:N ratio (root C:N). Leaf δ^13^C is a proxy of intrinsic water use efficiency (iWUE, Prieto et al., 2018; Ma et al., 2023). The Huber value reflects the mass investment in xylem area with respect to leaf area (Mencuccini et al., 2019); it is thus a measure of how species adjust their leaf and sapwood areas to water supply (Carter and White, 2009; Zhang et al., 2019) and is related to groundwater depth extraction (Carter and White, 2009). Thus, we used this trait as a proxy of the hydraulic architecture of the plants.

**Table 1:** List of the nine functional traits considered in this study, their abbreviations, units and their functional roles.

Trait	Abbreviation	Unit	Functional role	References
Leaf				
Leaf dry matter content	LDMC	mg g^-1^	Physical resistance and stress tolerance	Pérez-Harguindeguy et al., 2013; de la Riva et al., 2017
Specific leaf area	SLA	m^2^ kg^-1^	Leaf longevity, light capture and growth rate	Shipley and Vu, 2002
C:N content	Leaf C:N	unitless	Growth rate	Zhao et al., 2016; Zhang et al., 2020
Carbon isotopic composition	δ^13^C	‰	Gas exchange and water use efficiency	Prieto et al., 2018
Stem				
Stem dry matter content	SDMC	mg g^-1^	Physical resistance	de la Riva et al., 2018b
Huber value	H_v_	cm^2^ cm^-2^	Coordination of water transport and water loss	Mencuccini et al., 2019
Root				
Specific root area	SRA	m^2^ kg^-1^	Water and nutrients acquisition	de la Riva et al., 2021a
Average root diameter	Rdi	mm	Water and nutrients acquisition	Freschet et al., 2021
C:N content	Root C:N		Nutrients acquisition, root defence	Freschet et al., 2021

Modified from de Tomás Marín et al. (2023).

For leaf and stem measurements, two-year-old branches with fully expanded, sun-exposed mature leaves from five healthy adult individuals per species and community were sampled. A variable number of leaves were measured depending on the average leaf size of each species. For root measurements, fine roots (<2 mm diameter) from five individuals per species and community were collected within the first 20-30 cm of soil by excavating next to the plant stem base to ensure that roots belonged to the selected individual. Roots were stored fresh in a cooler after collection and taken to the lab on the same day, where they were rinsed with distilled water to eliminate adhered soil particles, and then frozen until they were measured. The Hv was measured at regional level, i.e. selecting a total of three individuals per species for all the species, except for *F. sylvatica* and *Q. pyrenaica*, for which we measured 5 individuals per community (de Tomás Marín et al., 2023).

Leaf and root material was scanned with an EPSON® V850 PRO scanner at a resolution of 600 dpi. Leaf area was measured from the scanned images using the ImageJ software (Schneider et al., 2012). Root area and diameter were measured from the scanned images using the WinRHIZO 2009 software (Regent Instruments Inc., Quebec, Canada). After scanning, roots were oven-dried at 60°C for 48h prior to measurements. Leaf carbon isotopic composition and C and N concentrations were measured simultaneously with a THERMO/Finnigan MAT V isotope ratio mass spectrometer, coupled to a THERMO Flash EA 1112 elemental analyzer via a THERMO/Finnigan Conflo IV- interface. Leaf carbon isotopic composition is expressed in the conventional delta notation (δ^13^C) relative to VPDB (Vienna PeeDee Belemnite standard). All plant material was collected, stored and processed following the protocols detailed by Pérez-Harguindeguy et al. (2013). For a detailed protocol of sample harvesting and trait measurements see also de Tomás Marín et al. (2023), de la Riva et al. (2016a) and de la Riva et al. (2016b).

### 2.4 Abiotic variables

In each selected plot, soil organic matter content (SOM), total soil N, soil nitrate and soil phosphate were determined from the top 15 cm of soil. Soil samples were collected with a hand trowel in three points in the plot, one of the samples corresponding to the center of the plot and the other two approximately 5 m above and below the center, and mixed in a plastic bag to homogenize the soil. Then soils were taken to the lab where they were dried at 50°C to constant weight (~72 h) and sieved at 2 mm before analyses. Additionally, slope, orientation, altitude and soil depth were also measured in the same plots (**Supplementary Material, Appendix S3, Table S3**). For the description of the methods employed to measure total soil N see Rodríguez-Calcerrada et al. (2019); for the methods employed to measure the rest of abiotic variables see de Tomás Marín et al. (2023).

### 2.5 Data analyses

As there is a great variety of ways in which plants combine a set of traits to achieve a successful performance (Díaz et al., 2016; Iozia et al., 2023), we focused on trait syndromes rather than on individual functional traits. To this end, we conducted a Principal Component Analysis (PCA) including the nine functional traits considered in this study, measured in 411 individuals belonging to the 19 sampled species, to describe trait trade-offs or trait syndromes (Albert et al., 2011). The first three principal components (PCs) had eigenvalues greater than one and represented three key dimensions of plant ecological strategies (**Supplementary Material**, **Appendix S4**, **Table S4**). That is, PC1, PC2 and PC3 reflected clear spectrums related to the Leaf Economics Spectrum (PC1, LES, Wright et al., 2004), Root Economics Spectrum (PC2, RES, de la Riva et al., 2018b) and hydraulic architecture (PC3, HyArq, Tyree and Ewers, 1991), respectively. We then used the scores of each individual in these three PCA axes to estimate the community-weighted moments and in further analyses.

#### 2.5.1 Community weighted moments along the environmental gradient

We calculated three informative moments related to the dominant trait values (mean) and the shape of the trait distribution (skewness and kurtosis) of the individual PCA scores for each of the three PC axes (LES, RES and HydArq) kept for analyses (see mathematical equations in **Supplementary Material, Appendix S5**). To scale up from species to community level, these moments were weighted by the relative abundance of each species in each community, resulting in the ‘community-weighted moments’ of the trait distributions. The community-weighted mean (CWM, Garnier et al., 2004) gives a representation of the dominant trait values within a community. This metric is directly related to Grime’s mass-ratio hypothesis (Grime, 1998) and considers that the traits of the most abundant species in the community have the largest influence in ecosystem processes (Bílá et al., 2014). The community-weighted skewness (CWS) quantifies the degree of asymmetry of the trait distribution within the community (Wieczynski et al., 2019; Gross et al., 2021). In our case, the CWS quantifies the degree of asymmetry of the individual values in the three spectrum axes (PC1, PC2 and PC3 scores) and reflects the presence of subordinate species within a community with trait values close to one of the extremes of the PC scores range (Le Bagousse-Pinguet et al., 2017). The community-weighted kurtosis (CWK) refers to the evenness of the distribution of individual values in the three spectrum axes (PC1, PC2 and PC3 scores) (Wieczynski et al., 2019; Gross et al., 2021), where a peaked distribution indicates low levels of diversity along the spectrums and a flat distribution indicates high levels of diversity.

To analyze the effect of the abiotic environment on the variation of the three community-weighted moments (CWM, CWS and CWK) of the different plant ecological dimensions in our communities (LES, RES and HyArq), we built multiple linear regressions models. The continuous abiotic variables (independent variables) and the discrete variable ‘community’ (factor) were introduced in the models as predictors and each of the three distribution moments of the selected PCs, separately, as response variables. The *lm* function from the base R package *stats* was used to perform the linear regression models analyses (R Core Team, 2021). To identify the best predictors, we applied the Akaike information criterion corrected for small sample size (AICc) on the full factorial model, i.e., including all the abiotic variables considered in the study (**Table 2**) and the communities. The selection of the best model (ΔAIC < 2) was performed using the function *dredge* from the *MuMIn* R package (Barton and Barton, 2015). The strength of the relationship between the predictors and the response variable for the selected models (lowest AICc) for each community-weighted moment and PC axis, was given by the adjusted R^2^, obtained with the *summary* function, also from the *stats* package (R Core Team, 2021). To assess the % of variance explained by each predictor in each model, we obtained the sum of squares of each predictor with the *Anova* function from the R package *car* (Fox and Weisberg, 2019) and calculated the proportion that the sum of squares of each variable was relative to the total sum of squares. To determine differences among the six communities for each community-weighted moment in each of PCs spectrum (LES, RES and HydArq), we ran one-way ANOVAs followed by *post-hoc* multiple pairwise comparisons (Tukey’s test), to detect differences between pairs of communities.

**Table 2:** Best-fitted linear regression models. Models are presented for each plant ecological strategy (LES, RES and HydArq) and for each of the Community Weighted moments (CWM, CWS, CWK).

Plant ecological strategy	Community weighted moments	Altitude	Orientation	Slope	Soil depth	Soil nitrate	Soil phosphate	Total soil N	SOM	Community	Adj. R^2^
LES	Mean		+ (4.3)		+ (1.7)					+ (60.4)	0.739
	Skewness		+ (4.0)							+ (61.6)	0.664
	Kurtosis									+ (48.9)	0.458
RES	Mean				+ (1.1)		- (0.9)			+ (84.5)	0.874
	Skewness				- (3.6)			+ (2.1)	+ (5.6)	+ (42.7)	0.662
	Kurtosis							+ (4.2)		+ (51.2)	0.515
HyArq	Mean	+ (4.7)	+ (3.6)		- (7.0)					+ (44.9)	0.626
	Skewness	- (4.1)			+ (8.1)					+ (23.7)	0.455
	Kurtosis									+ (52.8)	0.533

The best models were selected according to AICc values (ΔAICc<2). Slope direction (“+” or “-”) and the % of variance explained by each variable (value in brackets next to slope direction) are shown for variables included in the best fitting model. Abbreviations are as follows: LES, Leaf Economics Spectrum; RES, Root Economics Spectrum; HyArq, Hydraulic Architecture; SOM, Soil Organic Matter; Adj. R^2^, adjusted R^2^.

#### 2.5.2 Relative importance of intraspecific trait variability and species turnover

We determined the relative contribution of species turnover, i.e., the sum of the contributions of species occurrence and species abundance, and ITV to the overall change in the functional structure of our communities following the method developed by Lepš et al. (2011), using the function *traitflex.anova* implemented in the R software. For a detailed explanation of the method and mathematical calculations, please refer to the **Appendix S6** in the **Supplementary Materials** and to Lepš et al. (2011). Variance partitioning was estimated on the CWMs calculated for the first two PC axes (PC1 and PC2), which represent the LES and RES, respectively. We estimated two partitions in parallel, one with “community” as factor and the other with those abiotic variables selected by AICc in the multiple regression linear models (section 2.5.1), to assess the effects of the type of community and of the environment on the variation of CWMs, respectively. We could not carry out the partition for PC3 (HyArq) because Huber values were measured at the regional level, not at the community level.

All analyses were performed in R (version 4.1.2; R Core Team, 2021) interfaced with R Studio (RStudio Team, 2021).

## 3 Results

### 3.1 Trade-offs among traits

**Figure 2:**
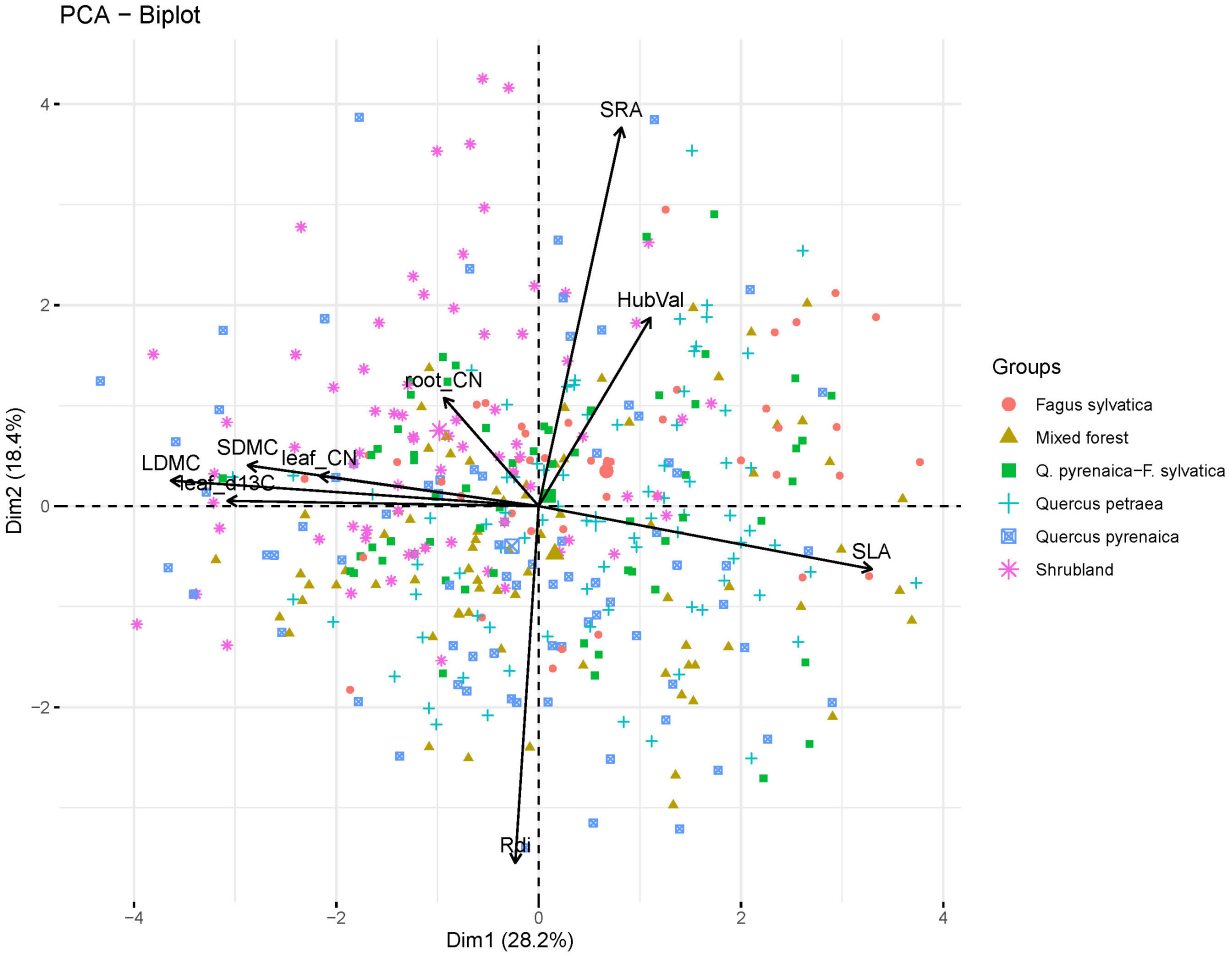
Principal Component Analysis (PCA). Results show the first two PC axes for the nine functional traits considered in this study measured on 411 individuals from 19 woody species (trait abbreviations are in **Table 1**; species list is in **Supplementary Material, Appendix S2, Table S2**). Symbols correspond to the 411 individuals. The type of community which observations belong to is shown with different symbols and colors.

According to the loadings on the PCA axes (**Supplementary Material**, **Appendix S4**, **Table S4**), the first three axes of variation (PC1, PC2 and PC3) were related to trait trade-offs within plant organs and thus to plant ecological dimensions (**Figure 2**). The first PCA axis accounted for 28.1% of the overall variation and was representative of the aboveground resource acquisition strategy or Leaf Economics Spectrum (LES, Wright et al., 2004). Positive values of the PC1 represented species with high SLA, indicative of fast resource uptake strategies at aboveground level, while in the opposite end of the spectrum were species with trait values indicative of the resource conservation strategy (high LDMC, leaf C:N, leaf δ^13^C and SDMC). The second PC axis explained 18.4% of the variability and was related to the Root Economics Spectrum (RES) (Prieto et al., 2015; de la Riva et al., 2018a). Within this axis, positive scores were associated to species with high SRA and negative scores to species with high Rdi. The third PC axis explained 13.7% of the variance and was associated to the Huber value, and thus to the plant’s hydraulic architecture (Mencuccini et al., 2019). Within this axis, positive scores were associated with species with high Huber values, i.e. conservative species.

### 3.2 Relationships among environmental variables and functional structure (community-weighted moments) and differences in the functional structure among communities

Overall, the models showed a high predictive power (**Table 2**) with all adjusted R^2^ values over 0.45. Our results showed that the type of community was the most important factor determining differences in the functional structure, with environmental factors having a secondary role (**Table 2**; **Appendix S7**). The effect of the different abiotic variables depended on the plant ecological dimension analyzed; altitude and orientation were relevant factors determining variation in the LES (aboveground level), while soil depth and nutrients were more important factors belowground, for RES, and HyArq (only soil depth in this case, **Table 2**).

**Figure 3:**
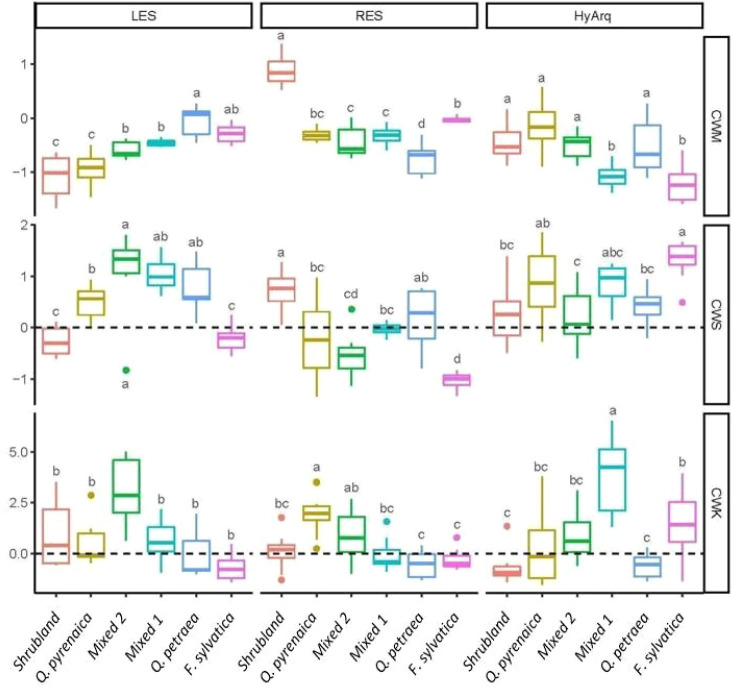
Boxplots of the community-weighted moments (CWM: community weighted mean, CWS: community weighted skewness and CWK: community weighted kurtosis) of each plant ecological strategy (LES, RES and HyArq, related to the principal components 1, 2 and 3, respectively; see materials and methods section for a detailed description of the different plant ecological strategies) for the six communities studied (see section ‘*2.2 Sampling design’* and **Supplementary Material, Appendix S2** for the composition of each community). LES, Leaf Economics Spectrum; RES, Root Economics Spectrum; HyArq, Hydraulic Architecture. The line inside the box represents the mean value, the box limits represent the SE and the whiskers represent the SD. Different letters indicate significant differences (*p* < 0.05) among communities. Community-weighted moments are significantly different from zero when its 95%-confidence interval does not overlap with zero (dashed lines).

Regarding variations in the functional structure among communities, we found clear differences in their community weighted moments related to the main axes of variation (**Figure 3**). The CWMs showed clear trends in relation to the position of the communities along the acquisitive-conservative axis of resource uptake. Mediterranean communities, i.e., *Shrubland* and *Q. pyrenaica* communities, had the lowest CWM scores in the LES (PC1 axis), i.e., these communities were composed by species with high tissue dry matter content and low SLA, and did not differ from each other, indicating a conservative resource-use strategy in both communities (**Figure 3**). In contrast, the Eurosiberian communities (dominated by either *Q. petraea* or *F. sylvatica*) showed the highest CWM scores in the LES axis (**Figure 3**); the two mixed communities showing intermediate CWM scores in the LES (**Figure 3**). In relation to the RES (**Figure 3**), the S*hrubland* community showed significantly higher CWM scores than the rest of the plant communities, indicating a more acquisitive resource use strategy belowground. *F. sylvatica* community had the second highest CWM scores, while *Q. petraea* community showed the lowest scores in the RES. Again, mixed communities (*Mixed* 1 and 2) showed intermediate CWM scores along the RES axis (**Figure 3**). With regard to the hydraulic strategy, we found an overall decreasing trend in CWM scores from Mediterranean to Eurosiberian communities (with the exception of *Q. petraea* community, which had similar CWM scores than Mediterranean communities; **Figure 3**).

We observed strong differences among communities on the community-weighted skewness (CWS) scores on the three plant ecological dimensions (LES, RES and HyArq), although we did not detect a clear pattern regarding the biogeographic origin of the communities (Mediterranean vs Eurosiberian). We found a high asymmetry in the scores’ distribution (high positive CWSs) in the two *Mixed* communities and in the *Q. petraea* community for the LES, in *Shrubland* community for the RES and in *F. sylvatica* community for HyArq. By contrast, *F. sylvatica* and the *Mixed* 2 (*F.sy-Q.py-Q.pe*) communities showed a high negative asymmetry (very low values) for the RES.

Similarly, community-weighted kurtosis (CWK) varied among communities depending on the plant ecological dimension considered. CWK values greater than 0 were found in the *Mixed 2* (*F.sy-Qpy-Qpe*) community for the LES, in the *Q. pyrenaica* community for the RES, and in the *Mixed 1* (*F.sy-Qpy*) community for the HyArq. By contrast, CWK scores for the *Shrubland*, *Q. petraea* and *F. sylvatica* communities did not differ from 0 for any plant ecological strategy (except for the HyArq axis in *F. sylvatica* community).

### 3.3 Contribution of ITV and species turnover to variation in the CWMs for the LES and RES ecological strategies

**Figure 4:**
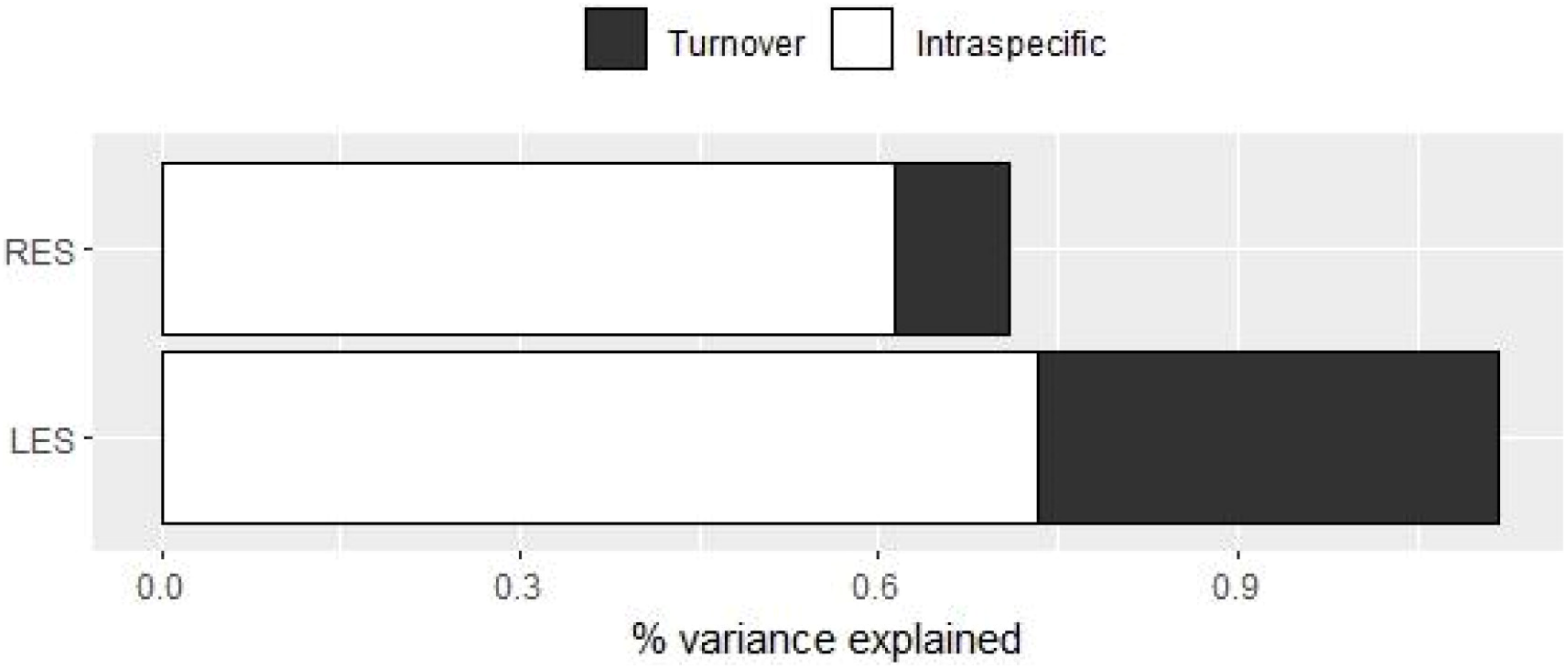
Decomposition of total variability in individual trade-offs among traits. LES corresponds to the trade-offs among the study traits related to the Leaf Economics Spectrum. The same applies for the RES in relation to the study traits related to the Root Economics Spectrum.

The percentage of variance explained by both ITV and species turnover was higher for the LES than for the RES (**Figure 4**; **Supplementary Material, Appendix S8, Table S8.1**). For both LES and RES, results showed a stronger influence of ITV than of species turnover in the CWMs’ variation among communities. This was especially evident for the RES, for which the effect of species turnover was particularly small compared to that of ITV: close to 61.3% of the variation was explained by ITV against 9.5% explained by turnover (**Figure 4**; **Supplementary Material, Appendix S8, Table S8.1**).

## 4 Discussion

### 4.1 Trait distribution and environmental effects on community assembly

We found large variation in the functional trait composition considered in this study. Our results showed that functional traits at community level (functional parameters) were mainly driven by differences among plant communities, while abiotic factors exerted a minor role on community weighted trait distributions. In this sub-Mediterranean ecosystem, we observed in a recent study (de la Riva et al., 2023) that environmental suitability explained species segregation better than their functional composition, being the six woody plant communities segregated spatially and functionally partly in relation to microenvironmental conditions, mostly soil depth at aboveground level, and aspect and soil nutrients at belowground level.

Overall, the CWMs showed a trend that was in line with the acquisitive-conservative axis of resource uptake (Reich, 2014; Díaz et al., 2016; de la Riva et al., 2016b) at aboveground level. Plant communities dominated by Mediterranean species (*Q. pyrenaica*, *Shrubland* and *Mixed* 1 and 2) are dominated by species with low SLA and SRA, and high LDMC, thick root diameters (Rdi), and high leaf and root C:N. These communities are found in harsh areas which are characterized by low soil depths with stony soils, steep slopes or high insolation (de la Riva et al., 2023; de Tomás Marín et al., 2023), conditions that probably limit water availability. In fact, this water shortage promoted the prevalence of communities with a conservative hydraulic architecture (i.e. water efficient with high leaf δ^13^C and Huber values), commonly associated with conservative water use strategies at the expense of a slower plant growth (Prieto et al., 2018; Mencuccini et al., 2019). On the other hand, communities dominated by Eurosiberian species (*Q. petraea* and *F. sylvatica* communities) are found in areas of Montejo with deeper soils —which likely increase soil water storage— and potentially lower evapotranspiration due to lower insolation. These communities had CWM values associated with faster growth and acquisitive resource-use strategies (i.e., higher SLA and SRA, and lower tissue dry matter content, Rdi, C:N ratios and Huber values).

When belowground strategies were considered, however, *Shrubland* community was the most acquisitive (high CWM) but also showed the highest positive skewness, indicating the presence of subordinate species with conservative strategies. This community occupies flat areas with shallow soils (and thus lower water availability) and high soil nutrient contents in Montejo (de la Riva et al., 2023). In these areas, greater aridity would promote the convergence of traits towards conservative strategies (Cornwell and Ackerly, 2009; de la Riva et al., 2018b; Carrascosa et al., 2023). Although shallow soils prevent the accumulation of big amounts of water, the flat terrain favors water accumulation in the soil surface during short periods. Under these conditions, with a high-enough nutrient concentration, fast resource-uptake belowground strategies would give plants a competitive advantage to absorb the water and nutrients accumulated in shallow soils (Ryel et al., 2008; Schenk, 2008; Querejeta et al., 2021), and to grow during short wet pulses (Querejeta et al., 2018; Carvajal et al., 2019; de la Riva et al., 2021b). Shrub species from Mediterranean arid environments are able to produce cheaper roots in terms of carbon investment to structural components, resulting in roots with high SRA and high absorptive efficiency (de la Riva et al., 2021b). Further, adaptation to low soil water availability during the summer months also explains the negative kurtosis of *Shrubland* for HyArq, indicative of an evenness in the distribution of trait values related to the plant water balance, promoting the coexistence of functionally contrasting species (Gross et al., 2021). Overall, these results suggest a niche segregation and/or complementarity in water-use strategies that could minimize competition and favor coexistence (Bello et al., 2019; Illuminati et al., 2022), which could lead to a more efficient use of water in the *Shrubland* community. These results are in line with a recent study (Illuminati et al., 2022), in which the authors found a clear niche segregation and complementarity in functional traits related to water-use strategy of species coexisting in a Mediterranean shrubland community, and suggested that the presence of species with contrasting water-use strategies may represent a key driver in the assembly of dryland communities.

At the other end of the environmental gradient (see also de la Riva et al., 2023), we found the *F. sylvatica* community with predominantly acquisitive strategies at the whole plant level. In fact, species with a profligate water-use strategy, such as *F. sylvatica* (de Tomás Marín et al., 2023), dominated the lowest areas of Montejo where deeper soils are found (de la Riva et al., 2023), which could explain the shift in the hydraulic dimension along the soil depth gradient better than soil nutrients. This profligate use of water and nutrients of *F. sylvatica* makes it a strong fast-growing species that promotes strong competitive exclusion (Meyer et al., 2003; Rodríguez-Calcerrada et al., 2011; Castaño-Santamaria et al., 2021). In this regard, the highest differences in the skewness values of the RES and HyArq dimensions imply that the asymmetric competition promoted by *F. sylvatica* selects for species with very different functional profiles to that of the dominant species (Le Bagousse-Pinguet et al., 2017). Thus, sub-dominant species seem to be able to buffer competition with *F. sylvatica* by displaying contrasting morphological resource uptake strategies, and possessing different hydrological architecture. As a matter of fact, deeper soils may favor the ecohydrological niche segregation among coexisting species (Silvertown et al., 2015), allowing species to buffer competition by the uptake of water from different soil depths.

In relation to the *Mixed* communities, overall and looking at the CWM, they occupied an intermediate position along the Economics Spectrum (LES, RES and HydArq) and along the environmental gradient in Montejo. These communities, however, exhibited an overall positive asymmetric distribution (CWS = 0.62 for *Mixed 1* and CWS = 0.27 for *Mixed 2*), i.e. a higher frequency of species with traits associated to conservative strategies (CWS > 0), and especially so for the LES dimension. These mixed communities assembled following a competitive hierarchy (de la Riva et al., 2023) where species with acquisitive strategies aboveground are excluded in favor of those with more conservative traits that confer higher competitive advantage in harsher environments (Gross et al., 2013; Carmona et al., 2019). This is further supported by the high kurtosis in both mixed communities for the LES and HyArq, respectively, reflecting a selection of conservative trait values under strong competition (Le Bagousse-Pinguet et al., 2017).

Further, we found that the PC scores for the three plant ecological dimensions were normally distributed across species in the *Q. pyrenaica* community, with a predominance of conservative traits. On the other hand, *Q. petraea* community showed the most conservative traits at root level, opposite to the aboveground level, and deviated from normality for the LES (CWS significantly different from 0), which suggests contrasting resource uptake strategies at leaf level in the less dominant species with regard to the dominant aboveground acquisitive profile of the community. Thus, and similarly to the mixed communities, the departure from normality in the LES dimension in the *Q. petraea* community (indicated by its positive CWS values) suggests a process of hierarchical competition (de la Riva et al., 2023) in which species with aboveground conservative strategies are favored. Interestingly, we observed a mismatch between CWM root and leaf strategies. This mismatch can be explained by the ability of *Q. petraea* to take up water from deep soil layers to cope with drought stress during dry periods (Zapater et al., 2011; Bello et al., 2019; Staszel et al., 2022). This habit would be consistent with a vertical decoupling between water and nutrient uptake (Querejeta et al., 2021), explaining its conservative fine root traits in the topsoil (<20 cm) associated with a lower capacity for nutrient uptake.

To the best of our knowledge, our study is the first one that incorporates the use of community-weighted moments to disentangle the mechanisms and processes driving community assembly in a sub-Mediterranean ecotone. In this regard, the use of community-weighted moments of a trait distribution allowed us to get a deeper insight into the mechanisms driving community assembly in this sub-Mediterranean forest that would be otherwise overlooked, supporting the need to use metrics-based indices beyond the mean for a complete understanding of the underlying assembly patterns of plant communities (Enquist et al., 2015; Le Bagousse-Pinguet et al., 2017; Wieczynski et al., 2019; Guerin et al., 2022).

### 4.2 Disentangling the role of species turnover and intraspecific variability in shaping the trait distribution between communities

Our results from the analysis of variance decomposition showed that variations in community functional traits along Montejo were mainly explained by changes in intraspecific trait variability (ITV above 60%) rather than by species turnover, in line with previous findings (Jung et al., 2014; Siefert and Ritchie, 2016; Niu et al., 2020; Guo et al., 2022; or Spitzer et al., 2023) but in sharp contrast with others (de Bello et al., 2011; Pérez-Ramos et al., 2012; Kichenin et al., 2013; de la Riva et al., 2016a; Weemstra et al., 2021). The contrasting results found in the literature suggest that the relative contribution of interspecific vs intraspecific variability to the functional structure among plant communities vary with the spatial scale considered and the range of environmental variation. For instance, in this study, 17 out of the 19 species occurred in at least two communities if not more, which could explain the low contribution of species turnover. These results add further support to the hypothesis that ITV gains importance at smaller spatial scales (Lajoie and Vellend, 2015; Siefert et al., 2015; Pichon et al., 2022), when environmental conditions are less variable and interspecific trait variation is low (Cordlandwehr et al., 2013; Petruzzellis et al., 2017). Our results add up to the increasing evidence of the preponderant role that above- and belowground ITV plays on community assembly, suggesting that, in transitional areas such as ecotones, species are not solely filtered based on their mean trait values but also by their trait variability (Siefert, 2012). In fact, ITV is higher across the whole forest than at the community level in the Hayedo de Montejo (de la Riva et al., 2023), supporting the notion that adaptive shifts in traits within species allow them to establish in different communities along transitional areas.

Despite a majority of studies focusing on aboveground traits (e.g. Lepš et al., 2011; Pérez-Ramos et al., 2012; Siefert, 2012; Kichenin et al., 2013; Jung et al., 2014; Siefert et al., 2015; Siefert and Ritchie, 2016; Henn et al., 2018; Niu et al., 2020; Chelli et al., 2021; Guo et al., 2022; Pichon et al., 2022), evidence showing the contribution of belowground intraspecific trait variability to community assembly is starting to accumulate (de la Riva et al., 2016a; Navarro-Fernández et al., 2016; Weemstra et al., 2021; Hogan et al., 2023; Spitzer et al., 2023). In this study we observed that the extent and sources of variation of root traits followed a similar pattern to those of leaves, even with a relatively higher importance of ITV for roots than for leaves (**Figure 4**; **Supplementary Material, Appendix S8, Table S8.1**). A recent study showed that ITV explained the mean trait variation of morphological root traits rather than of chemical root traits (Spitzer et al., 2023), while previous studies carried out in Mediterranean woody communities showed higher ITV for the number of arbuscular mycorrhizas vesicles than for SRL (Navarro-Fernández et al., 2016). This indicates that ITV differs between root traits, as found at the aboveground level (Lepš et al., 2011; Kichenin et al., 2013). Considering root trait syndromes at unified dimensions help to depict the role of ITV vs species turnover in community assembly, because traits co-vary from trade-offs between different plant functions, producing syndromes that influence species’ fitness (Albert et al., 2011).

## 5 Conclusions

**Figure 5:**
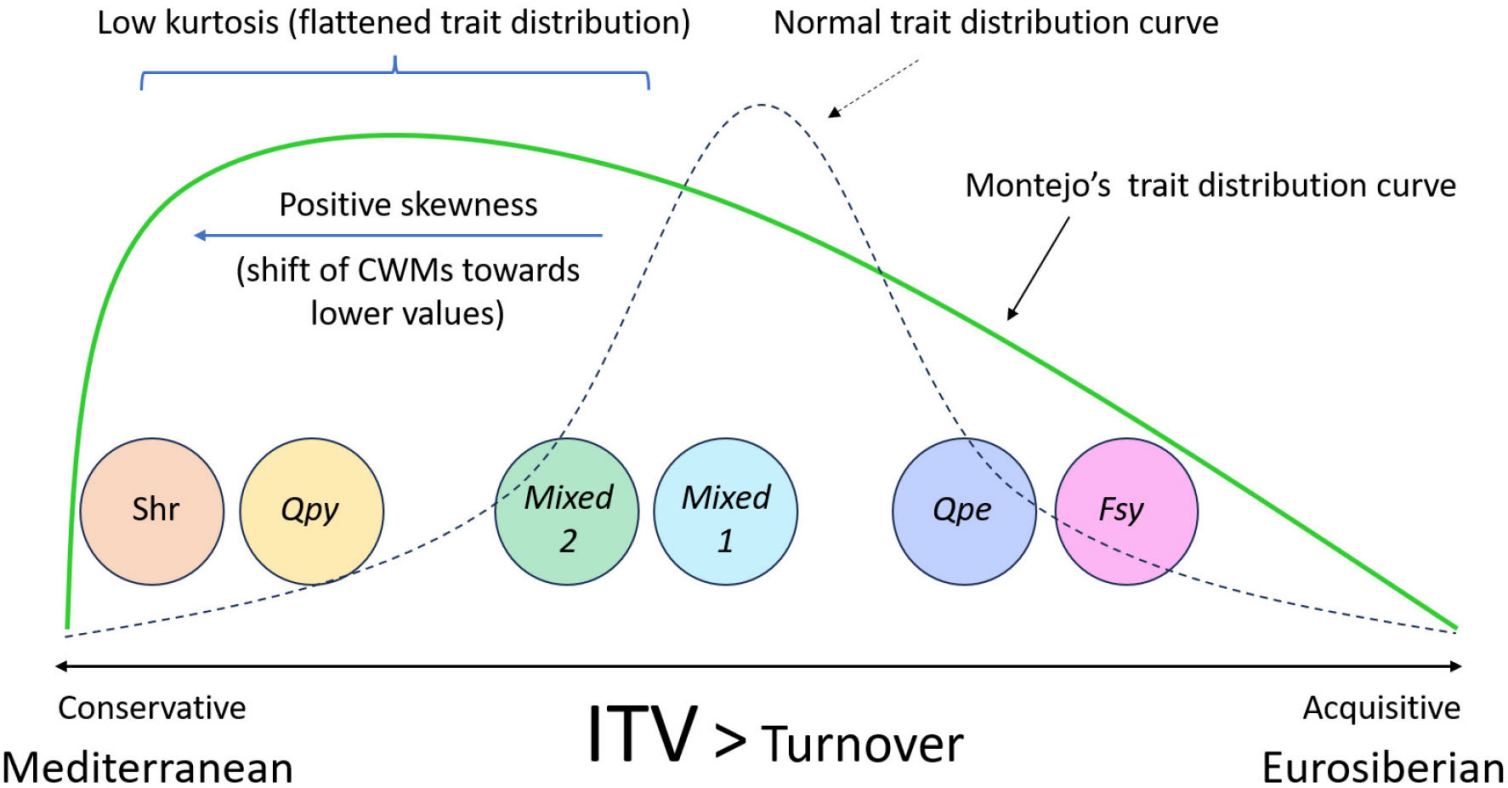
Schematic representation of the main findings of this study. Overall, the communities in Montejo spread along the conservative-acquisitive axis of resource use, partly matching their biogeographic origin (Mediterranean vs Eurosiberian). The positive skewness indicates a general shift of the Montejo forest towards more conservative trait values. The low kurtosis, shown by a flattened trait distribution curve, suggests the coexistence of different plant ecological strategies. Furhtermore, the relative contribution of ITV to the changes in the functional structure of Montejo is much higher than that of turnover, both at above- and belowground levels. Additionally, the type of community is the most relevant factor determining differences in the functinoal structure among communities, with environmental variables having a minor role. Shr, Shrubland community; Qpy, *Q. pyrenaica*-dominated community; *Mixed 1*, community co-dominated by *Fagus sylvatica* and *Q. pyrenaica*; *Mixed 2*, community co-dominated by *F.* sylvatica, *Q. pyrenaica* and *Q. petraea*; Qpe: *Q. petraea*-dominated community; Fsy, *F. sylvatica*-dominated community; ITV, Intraspecific Trait Variability.

In this study, we discerned the assembly patterns of woody plant communities in a sub-Mediterranean forest, using a whole-plant, trait-based approach. We observed a high number of strategies in the forest, reflecting coordination in the change of above- and belowground traits, with the different communities spreading along the acquisitive-conservative axis of resource-use, partly matching their Eurosiberian vs Mediterranean nature, respectively. Additionally, we showed that ITV is a highly relevant factor that should be incorporated when analyzing functional changes and assembly patterns in plant communities, both above- and belowground, especially at small spatial scales. We also found slight relationships between changes in trait syndromes and topographic and soil factors. This is probably due to the small spatial scale and sampling area of the study and to the potential biotic interactions occurring among the different plant species, which may prevent us from detecting stronger functional responses to environmental conditions (See **Figure 5** for a conceptual illustration of the main findings of the study).

Altogether, these findings represent an important step towards a more general understanding of the processes driving the assembly and functional composition of sub-Mediterranean plant communities. Our data support the notion that ecotones are fragile regions where minor environmental shifts may result in abrupt changes in ecosystem structure and composition. Thus, understanding which and how abiotic factors produce changes in the composition and functional structure of these transitional regions (e.g. species turnover vs intraspecific variability) may help to their protection in the face of global change. Our results set the ground for future research of community assembly processes and mechanisms of plant communities in these and other ecotone regions.

## Data availability statement

The raw data supporting the conclusions of this article will be made available by the authors, without undue reservation.

## Author contributions

ST: Data curation, Formal Analysis, Writing – original draft, Writing – review & editing. JG: Data curation, Formal Analysis, Writing – review & editing. JR-C: Writing – review & editing. IP: Conceptualization, Methodology, Writing – review & editing. ER: Conceptualization, Funding acquisition, Methodology, Resources, Supervision, Writing – review & editing.
