# Supplementary material for: Linking functional composition moments of the sub-Mediterranean ecotone with environmental drivers
Source: Front Plant Sci. 2023 Dec 8;14:1303022. doi: 10.3389/fpls.2023.1303022 (PMC10748396; doi:10.3389/fpls.2023.1303022)
Supplement: Supplementary file 1 [file DataSheet_1.docx]

**SUPPORTING INFORMATION**

**APPENDIX S1. Zonification of the study area.**


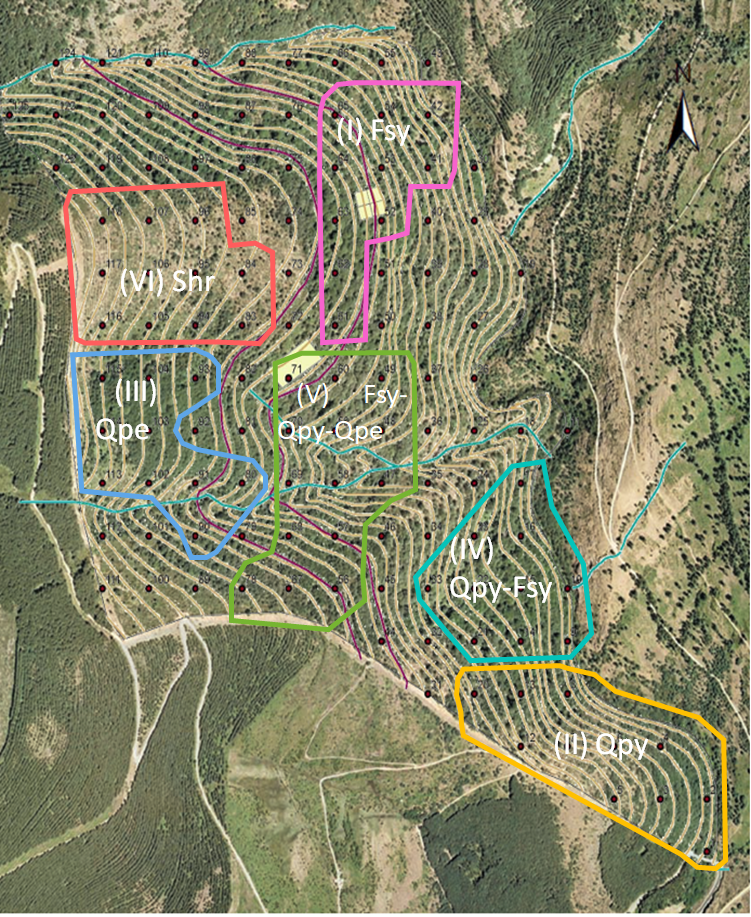


**Supplementary Figure S1**. Aerial photography of the study area. Montejo forest corresponds to the area occupied by the contour lines. Each polygon delimits a different community: (I) Fsy = *Fagus sylvatica* dominated community, (II) Qpy = *Quercus pyrenaica* dominated community, (III) Qpe = *Quercus petraea* dominated community (when >60% of the abundance corresponded to one of these three species, respectively), (IV) Qpy-Fsy = mixed forest 1 (transition areas between *F. sylvatica* and *Q. pyrenaica*), (V) Fsy-Qpy-Qpet = mixed forest 2 (transition area with co-dominance of *F. sylvatica*, *Q. pyrenaica* and *Q. petraea*) and (VI) Shr = shrubland community. Black dots indicate the center of each sampling plot in which the forest is divided.

**APPENDIX S2. List of study species and their relative abundances in each community where they were found.**

| **Species** |  | **Growth form** | **Relative abundance (%) per community** | | | | | |
| --- | --- | --- | --- | --- | --- | --- | --- | --- |
|  |  |  | I | II | III | IV | V | VI |
| *Adenocarpus hispanicus* |  | Shrub | 0.42 | 1.65 | 1.01 | 0.69 | 2.80 | 10.27 |
| *Crataegus monogyna* |  | Shrub | 1.58 | 6.77 | 1.03 | 2.61 | 4.18 | 0.99 |
| *Cytisus purgans* |  | Shrub | 0.00 | 0.00 | 0.00 | 0.00 | 0.00 | 3.73 |
| *Cytisus scoparius* |  | Shrub | 0.00 | 5.30 | 0.80 | 0.23 | 2.19 | 0.59 |
| *Erica arborea* |  | Shrub | 0.00 | 11.76 | 0.99 | 1.13 | 2.41 | 4.27 |
| *Erica australis* |  | Shrub | 0.00 | 0.48 | 0.00 | 0.00 | 0.00 | 0.49 |
| *Fagus sylvatica* |  | Tree | 61.27 | 8.59 | 27.83 | 38.57 | 16.66 | 0.00 |
| *Genista florida* |  | Shrub | 0.06 | 4.29 | 0.63 | 0.64 | 2.53 | 3.23 |
| *Hedera helix* |  | Liana | 1.24 | 1.03 | 0.90 | 1.38 | 1.34 | 0.00 |
| *Ilex aquifolium* |  | Arborescent tree | 11.47 | 0.91 | 17.57 | 2.42 | 5.43 | 1.66 |
| *Juniperus communis* |  | Shrub | 0.00 | 1.14 | 1.20 | 0.03 | 0.12 | 8.49 |
| *Lavandula stoechas* |  | Shrub | 0.00 | 1.18 | 0.64 | 0.74 | 0.34 | 11.92 |
| *Prunus avium* |  | Tree | 0.00 | 0.02 | 0.32 | 3.31 | 1.55 | 2.80 |
| *Quercus petraea* |  | Tree | 23.57 | 0.00 | 45.14 | 1.01 | 20.27 | 8.51 |
| *Quercus pyrenaica* |  | Tree | 0.35 | 51.16 | 0.65 | 45.90 | 36.36 | 16.42 |
| *Rosa sp.* |  | Liana | 0.02 | 4.89 | 0.86 | 1.19 | 2.31 | 11.99 |
| *Rubus sp.* |  | Liana | 0.02 | 0.55 | 0.01 | 0.15 | 0.19 | 1.75 |
| *Sorbus aria* |  | Tree | 0.00 | 0.00 | 0.22 | 0.00 | 0.81 | 6.25 |
| *Sorbus aucuparia* |  | Tree | 0.00 | 0.29 | 0.20 | 0.00 | 0.52 | 6.64 |

Table S2. List of species sampled for trait measurements and their relative abundance (expressed in % of linear cover) per community: (I) *Fagus sylvatica*, (II) *Quercus pyrenaica*, (III) *Quercus petraea* (when >60% of the abundance corresponded to one of these three species, respectively), (IV) mixed forest 1 (transition areas between *F. sylvatica* and *Q. pyrenaica*), (V) mixed forest 2 (transition area with codominance of *F. sylvatica*, *Q. pyrenaica* and *Q. petraea*) and (VI) Shrubland.

**APPENDIX S3. Abiotic variables considered in the study.**

Table S3. List of abiotic variables considered in this study, with values specified across the plots from the Forest Inventories and communities from where they were measured. See Supplementary Figure S1 for description of communities I through VI.

| Community | plot ID | Slope (°) | Orientation (°N) | Altitude (m) | Soil depth (m) | SOM (% dry weight) | Soil Nitrate (μg/g dry soil) | Soil Phosphate (μg/g dry soil) | Total soil N (%) |
| --- | --- | --- | --- | --- | --- | --- | --- | --- | --- |
| II | 1 | 19 | 103 | 1253 | 1.15 | 14.21 | 0.79 | 5.59 | 0.259 |
| II | 2 | 19 | 77 | 1257 | 0.89 | 13.16 | 0.96 | 5.32 | 0.315 |
| II | 3 | 25 | 83 | 1297 | 0.77 | 14.09 | 2.52 | 31.45 | 0.188 |
| II | 4 | 24 | 29 | 1277 | 0.75 | 17.04 | 0.65 | 3.41 | 0.32 |
| II | 5 | 19 | 65 | 1332 | 0.63 | 15.41 | 1.06 | 11.31 | 0.271 |
| II | 6 | 29 | 29 | 1302 | 0.42 | 22.72 | 2.44 | 15.66 | 0.315 |
| II | 7 | 31 | 20 | 1323 | 0.64 | 17.67 | 1.18 | 5.05 | 0.624 |
| II | 8 | 28 | 74 | 1277 | 1.09 | 12.96 | 1.02 | 7.77 | 0.152 |
| IV | 9 | 22 | 89 | 1264 | 0.99 | 14.33 | 0.96 | 21.92 | 0.271 |
| IV | 10 | 15 | 77 | 1261 | 0.46 | 7.54 | 0.58 | 11.85 | 0.331 |
| II | 12 | 23 | 50 | 1354 | 0.81 | 18.96 | 9.35 | 33.35 | 0.321 |
| IV | 14 | 23 | 66 | 1311 | 0.75 | 16.97 | 0.88 | 5.86 | 0.363 |
| IV | 15 | 16 | 77 | 1295 | 0.72 | 10.32 | 0.7 | 13.48 | 0.253 |
| IV | 16 | 31 | 80 | 1286 | 1.01 | 13.33 | 0.57 | 7.5 | 0.534 |
| IV | 17 | 28 | 98 | 1283 | 0.84 | 8.84 | 0.58 | 6.95 | 0.301 |
| II | 20 | 27 | 70 | 1370 | 0.82 | 14.32 | 0.88 | 11.31 | 0.392 |
| IV | 21 | 28 | 50 | 1348 | 1.05 | 13.59 | 0.95 | 9.95 | 0.323 |
| IV | 22 | 14 | 56 | 1322 | 1.18 | 13.4 | 1.17 | 14.3 | 0.317 |
| IV | 23 | 27 | 97 | 1321 | 0.82 | 15.2 | 0.81 | 10.22 | 0.462 |
| IV | 33 | 27 | 69 | 1359 | 1.18 | 12.77 | 0.98 | 8.04 | 0.295 |
| I | 41 | 22 | 100 | 1337 | 0.69 | 13.97 | 0.85 | 8.86 | 0.289 |
| I | 42 | 27 | 41 | 1305 | 1.01 | 15.52 | 0.71 | 3.96 | 0.237 |
| V | 48 | 18 | 173 | 1359 | 0.6 | 17.93 | 1.7 | 15.12 | 0.35 |
| V | 49 | 21 | 95 | 1379 | 0.81 | 10.38 | 1.59 | 17.02 | 0.46 |
| I | 52 | 27 | 96 | 1389 | 1.22 | 12.7 | 1.07 | 3.96 | 0.366 |
| I | 53 | 27 | 59 | 1377 | 1.01 | 21.76 | 2.09 | 21.1 | 0.487 |
| I | 54 | 30 | 68 | 1354 | 0.71 | 28.8 | 1.36 | 8.86 | 0.269 |
| V | 58 | 22 | 156 | 1369 | 0.33 | 10.82 | 0.37 | 6.68 | 0.365 |
| V | 59 | 17 | 140 | 1381 | 1.02 | 12.42 | 0.81 | 3.96 | 0.292 |
| V | 60 | 12 | 138 | 1400 | 1.38 | 22.91 | 6.78 | 22.47 | 0.45 |
| I | 61 | 12 | 110 | 1411 | 0.83 | 31.83 | 13.01 | 20.56 | 0.484 |
| I | 62 | 25 | 125 | 1418 | 0.8 | 22.74 | 1.97 | 23.28 | 0.319 |
| I | 63 | 18 | 85 | 1424 | 1.5 | 15.69 | 1.79 | 6.41 | 0.388 |
| I | 64 | 19 | 67 | 1415 | 1.12 | 29.79 | 1.23 | 31.17 | 0.432 |
| I | 65 | 20 | 41 | 1391 | 1.5 | 19.66 | 3.62 | 27.91 | 0.512 |
| V | 67 | 19 | 36 | 1450 | 0.83 | 26.49 | 6.36 | 20.29 | 0.424 |
| V | 68 | 20 | 20 | 1419 | 0.71 | 25.14 | 3.22 | 15.93 | 0.302 |
| V | 69 | 25 | 143 | 1394 | 0.56 | 14.18 | 10.84 | 31.72 | 0.317 |
| V | 70 | 15 | 100 | 1394 | 0.97 | 13.79 | 0.84 | 7.5 | 0.407 |
| V | 71 | 13 | 149 | 1416 | 1.16 | 17.79 | 1.18 | 13.21 | 0.544 |
| III | 80 | 21 | 145 | 1421 | 0.94 | 19.74 | 2.59 | 13.48 | 0.422 |
| VI | 83 | 12 | 134 | 1445 | 1.34 | 30.86 | 2.35 | 14.3 | 0.747 |
| VI | 84 | 9 | 131 | 1459 | 1.29 | 24.98 | 1.84 | 9.4 | 0.459 |
| III | 90 | 21 | 36 | 1450 | 0.85 | 21.25 | 1.79 | 15.66 | 0.343 |
| III | 91 | 23 | 156 | 1442 | 1.13 | 11.1 | 0.86 | 6.41 | 0.33 |
| III | 93 | 17 | 104 | 1453 | 0.89 | 11.85 | 0.84 | 5.32 | 0.386 |
| VI | 95 | 16 | 107 | 1478 | 0.66 | 28.57 | 2.48 | 21.1 | 0.917 |
| VI | 96 | 13 | 70 | 1480 | 1.04 | 52.63 | 7.54 | 78.8 | 0.477 |
| III | 102 | 20 | 155 | 1474 | 0.73 | 13.51 | 0.66 | 3.41 | 0.523 |
| III | 103 | 17 | 82 | 1484 | 0.64 | 17.8 | 0.92 | 5.59 | 0.418 |
| III | 104 | 14 | 89 | 1482 | 0.83 | 17.62 | 0.95 | 6.68 | 0.418 |
| VI | 105 | 17 | 120 | 1490 | 1.25 | 19.69 | 2.41 | 16.75 | 1.084 |
| VI | 106 | 14 | 103 | 1504 | 0.69 | 24.56 | 2.57 | 40.7 | 0.648 |
| VI | 107 | 12 | 63 | 1504 | 0.43 | 29.36 | 1.07 | 22.19 | 0.494 |
| III | 113 | 29 | 136 | 1507 | 0.61 | 22.06 | 1.67 | 11.31 | 0.296 |
| III | 114 | 20 | 91 | 1517 | 0.95 | 25.37 | 6.11 | 25.46 | 0.532 |
| III | 115 | 18 | 75 | 1509 | 0.86 | 20.86 | 13.82 | 27.09 | 0.614 |
| VI | 116 | 22 | 123 | 1519 | 0.86 | 31.2 | 5.1 | 59.21 | 0.937 |
| VI | 117 | 16 | 99 | 1533 | 0.45 | 27.43 | 2.38 | 22.19 | 0.334 |
| VI | 118 | 20 | 52 | 1528 | 0.54 | 26.36 | 7.08 | 31.72 | 0.705 |

**APPENDIX S4. Results from the PCA carried out for the nine above- and belowground functional traits measured on 411 individuals from the 19 woody species from which traits were measured.**

Table S4. Loadings of all the principal components (PC) of the PCA carried out for the nine above- and belowground functional traits measured on 411 individuals of 19 woody species. It is also shown the standard deviation, proportion of variance, cumulative variance and eigenvalues for each PC. See full name, units and functional meaning of the study traits in the main text (table 1).

| Trait | PC1 | PC2 | PC3 | PC4 | PC5 | PC6 | PC7 | PC8 | PC9 |
| --- | --- | --- | --- | --- | --- | --- | --- | --- | --- |
| LDMC | -0.52 | 0.04 | -0.22 | 0.01 | -0.21 | -0.06 | 0.33 | -0.62 | -0.38 |
| SLA | 0.47 | -0.11 | -0.41 | 0.16 | -0.14 | -0.14 | 0.26 | 0.32 | -0.60 |
| Leaf δ^13^C | -0.44 | 0.01 | -0.01 | -0.27 | 0.56 | 0.32 | 0.18 | 0.43 | -0.31 |
| Leaf C:N | -0.31 | 0.05 | 0.46 | 0.36 | -0.57 | 0.16 | 0.17 | 0.42 | -0.07 |
| SDMC | -0.41 | 0.07 | -0.29 | -0.16 | -0.15 | -0.66 | -0.39 | 0.34 | 0.03 |
| H_v_ | 0.16 | 0.33 | 0.57 | -0.10 | 0.25 | -0.57 | 0.33 | -0.06 | -0.17 |
| SRA | 0.12 | 0.66 | 0.06 | -0.11 | -0.11 | 0.27 | -0.52 | -0.09 | -0.41 |
| Rdi | -0.03 | -0.63 | 0.39 | 0.07 | 0.10 | -0.08 | -0.47 | -0.16 | -0.44 |
| Root C:N | -0.13 | 0.19 | -0.13 | 0.85 | 0.43 | -0.09 | -0.13 | -0.04 | 0.02 |
| Standard deviation | 1.59 | 1.29 | 1.11 | 0.99 | 0.91 | 0.86 | 0.64 | 0.60 | 0.50 |
| Prop. Variance | 0.281 | 0.184 | 0.137 | 0.11 | 0.09 | 0.08 | 0.05 | 0.04 | 0.03 |
| Cumulative var. | 0.28 | 0.46 | 0.60 | 0.71 | 0.80 | 0.89 | 0.93 | 0.97 | 1.00 |
| Eigenvalue | 2.53 | 1.65 | 1.23 | 0.99 | 0.83 | 0.74 | 0.41 | 0.36 | 0.25 |
|  |  |  |  |  |  |  |  |  |  |

**APPENDIX S5. Calculations of community-weighted mean, skewness and kurtosis.**

Mean, skewness and kurtosis of the trait distributions were calculated as follows:

(Eq. 1)

CWS*_j_* = $\frac{\sum_{i}^{s_{j}} pij(xij-{{CWM}_{j})}^{3}}{{{CWV}_{j}}^{3/2}}$ (Eq. 2)

CWK*_j_* = $\frac{\sum_{i}^{s_{j}} pij(xij-{{CWM}_{j})}^{4}}{{{CWV}_{j}}^{2}}$ -3 (Eq. 3)

where CWM*_j_*, CWS*_j_* and CWK*_j_* are community-weighted mean, skewness and kurtosis of the community *j*, respectively; $s_{j}$ is the number of species with available trait values in the community *j*; $pij$ is the relative abundance of species *i* in community *j;* and $xij$is the mean trait value of species *i* in community *j*.

**APPENDIX S6. Calculation of the relative contribution of species turnover and intraspecific variability to variation in the functional structure of the different communities in Montejo.**

To estimate the relative contributions of species turnover and intraspecific variability to variations in the functional structure of our communities, we first calculated the three CWMs parameters proposed by Lepš et al. (2011): 1) ‘*specific’* average, which corresponds to the CWM calculated using averaged trait values from each species in each site separately, and whose variation can be produced by both species turnover and intraspecific variability; 2) *‘fixed’* trait values, using the averaged trait values of each species independently of the site (i.e. site-independent trait values), whose variation can be only due to species turnover; and 3) *‘intraspecific variability’* trait values, obtained from the difference between *‘specific’* and *‘fixed’* trait averages, which informs about the changes in trait values only due to intraspecific variability:

intraspecific variability = specific parameter – fixed parameter

Then, to quantify how much variability is explained by each individual component (i.e., species turnover and intraspecific variability) we used the method proposed by Lepš et al. (2011) based on the Sum of Squares decomposition. This method is implemented by means of the *traitflex.anova* function for the R software (R Core Team, 2021). For mathematical details see Lepš et al. (2011).

**APPENDIX S7. Percentage of variance explained by the independent variables included in each of the best-fitted linear regression models for each plant ecological dimension and community-weighted moment.**

Table S7.1. Percentage of variance explained by each of the independent variables included in the best-fitted linear regression model of each community-weighted moment for each plant ecological dimension.

| Model predictors | CWM LES | CWM RES | CWM HyArq | CWS LES | CWS RES | CWS HyArq | CWK LES | CWK RES | CWK HyArq |
| --- | --- | --- | --- | --- | --- | --- | --- | --- | --- |
| (Intercept) | 11.4 | 0.5 | 6.4 | 5.0 | 10.8 | 5.2 | 3.0 | 5.0 | 7.9 |
| Community | 60.4 | 84.5 | 44.9 | 61.6 | 42.7 | 23.7 | 48.9 | 51.2 | 52.8 |
| Altitude |  |  | 4.7 |  |  | 4.1 |  |  |  |
| Orientation |  |  | 3.6 | 4.0 |  |  |  |  |  |
| Slope |  |  |  |  |  |  |  |  |  |
| Soil depth | 1.7 | 1.1 | 7.0 |  | 3.6 | 8.1 |  |  |  |
| Soil nitrate |  |  |  |  |  |  |  |  |  |
| Soil phosphate |  | 0.9 |  |  |  |  |  |  |  |
| Total soil N |  |  |  |  | 2.1 |  |  | 4.2 |  |
| SOM |  |  |  |  | 5.6 |  |  |  |  |
| Residuals | 22.3 | 13.0 | 33.3 | 29.4 | 35.2 | 58.8 | 48.1 | 39.6 | 39.4 |

**APPENDIX S8. Contribution of species occurrence, abundance and intraspecific variability to changes in the LES and RES axes.**

Table S8.1. Relative contributions of species occurrences, abundances and intraspecific variability to the total variation of the LES and the RES axes.

|  | Turnover | Intraspec. | Covariation | Total |
| --- | --- | --- | --- | --- |
| LES | 0.3856 | 0.7322 | -0.1178 | 1 |
| RES | 0.09514 | 0.6128 | 0.2921 | 1 |
